# Total laryngectomy vs. non-surgical organ preservation in advanced laryngeal cancer: a metanalysis

**DOI:** 10.1016/j.bjorl.2024.101404

**Published:** 2024-02-21

**Authors:** Elio Gilberto Pfuetzenreiter Jr., Gabriela Feltrini Ferreron, Julia Zumerkorn Sadka, Ana Beatriz Pádua de Souza, Leandro Luongo Matos, Luiz Paulo Kowalski, Rogério Aparecido Dedivitis

**Affiliations:** a Fundação Lusíada (UNILUS), Departamento de Cirurgia, Santos, SP, Brazil; b Universidade de São Paulo (FMUSP), Faculdade de Medicina, Divisão de Cirurgia de Cabeça e Pescoço, São Paulo, SP, Brazil

**Keywords:** Laryngectomy, Organ preservation treatment, Metanalysis, Larynx cancer

## Abstract

**Objective:**

To compare the oncological results and the functional outcomes of patients undergoing Total Laryngectomy (TL) with the non-surgical treatment (organ preservation protocol) in the treatment of advanced laryngeal carcinomas through systematic review and meta-analysis.

**Methods:**

A literature survey strategy was employed in order to perform a systematic review of the available evidence. Success rate and functional outomes after oncological treatment of patients with advanced laryngeal carcinomas was evaluated through systematic review and metanalysis, comparing TL and organ preservation protocol.

**Results:**

The surgical treatment was associated with better survival outcomes. When stratifying by T stage, while patients with T4 staging have less risk of mortality with TL, there is no difference between the different treatments for patients with T3 tumors. Surgery is related to a lower chance of recurrence, late dysphagia and feeding tube dependence.

**Conclusion:**

Patients with T4 tumors should undergo TL as their treatment of choice. For patients with T3 tumors, there is no differences on the risk of mortality according to the therapeutic option, however, there is a greater chance of recurrence and dysphagia when surgery is not performed.

## Highlights

• Patients with advanced laryngeal SCC have better surviving rates when submitted to TL as the first-choice treatment.
• Patients with T4 tumors should have TL as their treatment of choice.
• T3 tumors have similar survival rates with both treatments. Higher recurrence and worse disease-free survival with NST.
• Dysphagia and feeding tube dependence are more likely to happen when using organ preservation treatment.

## Introduction

Brazil has one of the greatest incidences of laryngeal cancer in the world, with 7.700 new cases a year.^1^ According to the American Joint Committee on Cancer, advanced cases present stage III and IV, in which are included locally advanced tumors and/or lymph node or distant metastases.^2^

The treatment of laryngeal cancer can result in very morbid consequences and lead to a significant deterioration in the patient’s quality of life. Despite being the standard procedure for the treatment of the advanced cases for almost 150-years, Total Laryngectomy (TL) is feared by many patients due to the stigma of definitive tracheostomy and voice loss. This often leads to the choice of Non-Surgical Treatments (NST).^3^

Thus, many centers have adopted different measures aiming to avoid total laryngectomy, and directed the treatment to the organ preservation options, hoping to maintain patient’s survival while preserving the organ. However, it has been suggested that the increased use of organ preservation strategies in advanced cases could be leading to poor survival rates.^4^ However, there is no well-defined consensus on how the treatment for the advanced laryngeal carcinoma cases may change the prognosis of patients. Nowadays, the best option is not defined.^5^

This study has the objective to do a systematic review and meta-analysis to compare: 1) The oncological results of patients undergoing TL with the non-surgical treatment (organ preservation protocol) in the treatment of advanced laryngeal carcinomas; 2) The functional outcomes of patients undergoing TL with the Non-Surgical Treatment (NST) in the treatment of advanced laryngeal carcinomas.

## Methods

This study was approved by the ethics committee, number 144/18. The study was made according the PRISMA Statement.^6^

### Identification and selection of the studies

A literature survey strategy was employed in order to perform a systematic review of the available evidence. This included research into the electronic database Medline in Pubmed (www.ncbi.nlm.nih.gov/pubmed), Scielo, Lilacs, Cochrane and EMBASE on June 2023, using the search strategy with the key words “(*laryngeal neoplasms OR laryngeal cancer OR laryngeal carcinoma) AND (laryngectomy) AND (organ-sparing treatments OR radiotherapy OR chemoradiotherapy OR organ preservation)*”. Results from 1992 to May 2023 were used. References of the selected studies to screen material not found in the electronic searches were also consulted through manual search.

Two reviewers independently screened the titles and abstracts for initial relevance evaluation. For all initially retrieved articles, if either reviewer considered any titles or abstracts meeting the eligibility criteria, their full-text form were then obtained. The quality and bias risk of the papers were critically appraised separately by the 2 reviewers. Any disagreements of extracted data were resolved through discussion between the two reviewers to reach a consensus or by consulting a third reviewer if necessary. Then, the data was collected from the papers which had appropriate information for analysis.

### Inclusion and exclusion criteria

The inclusion criteria were: (a) Clinical studies comparing the healing effects between TL and Radiotherapy (RT) or Chemo-Radiotherapy (CRT); (b) Patients with advanced laryngeal Squamous Cell Carcinoma (SCC) without previous treatment; (c) The cancer could be from any part of the larynx (glottis, supraglottis or infraglottis); (d) Original articles that could provide sufficient information for a metanalysis; and (e) Publications in English, Portuguese or Spanish languages.

The following exclusion criteria were used: (a) Studies that had patients undergoing partial laryngectomies; (b) Studies that had patients with tumors originating in hypopharynx; (c) Patients with distant metastases or undergoing palliative treatments; (d) Studies that lacked information from the Hazard Ratio and Confidence Intervals for the analysis of survival rates.

### Outcome evaluation

Success rate after oncological treatment of patients with advanced laryngeal carcinomas. The items evaluated for cancer outcomes were overall survival, disease-specific survival, disease-free survival, and recurrence rates. Long-term dysphagia and the need for a definitive feeding tube were also evaluated.

Hazard ratio and intervals of confidence were collected and used for the survival analyses, and the absolute number of cases and mean differences were used for the other items.

### Evidence level and methodological quality

The quality of the selected studies was analyzed in detail in order to evaluate the strength of their evidence and the validity of their inclusion in the present paper. The classification of the recommendation grade, which corresponds to the scientific strength of the study, was based on the National Health Service Centre for Evidence-Based Medicine and the Newcastle-Ottawa scale for the assessment of the quality of nonrandomized studies in meta-analyses.^7^, ^8^

### Statistical analysis

The measures of effectiveness or damage expressed through absolute values were analyzed by means of absolute risk reduction, under the Confidence Interval of 95%. The Number Necessary to Treat (NNT) values and the Number Necessary to Harm (NNH) values were respectively calculated for all statistically significant results. The continuous data were analyzed regarding their averages and standard deviations. The difference between the weighted average of the groups was employed for the analysis.

The metanalyses were made using the software Review Manager 5.3 (The Cochrane Collaboration), using the Hazard ratios and Confidence Interval for the survival analyses, and a *p*-value < 0.05 was considered statistically significant.

Heterogeneity between studies was estimated by Cochran Q-test and measured by I^2^ value (inconsistency index), and it was considered present when I^2^ > 50%. In this situation, aleatory effect models were adopted. On the contrary, a fixed effect model (inverse-variance model) was used. Publication bias was only assessed if there were more than 10 studies in the analysis.

## Results

**Figure 1:**
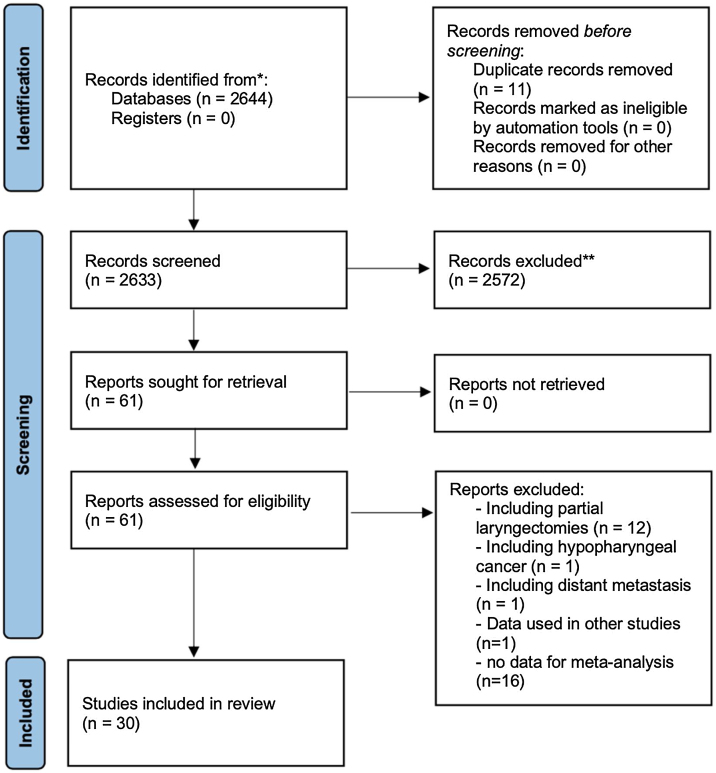
Flowchart of the electronic survey.

Fig. 1 shows how the article selection process was performed. In the initial search, 2115 results appeared on Pubmed, 15 on Scielo, 114 on Cochrane and 400 on EMBASE. A total of 31 studies was selected, all retrospective.^9^, ^10^, ^11^, ^12^, ^13^, ^14^, ^15^, ^16^, ^17^, ^18^, ^19^, ^20^, ^21^, ^22^, ^23^, ^24^, ^25^, ^26^, ^27^, ^28^, ^29^, ^30^, ^31^, ^32^, ^33^, ^34^, ^35^, ^36^, ^37^, ^38^, ^39^ Unfortunately there are not any prospective trials which could be used according to inclusion and exclusion criteria (used partial laryngectomies, did not use the Hazard ratios, had hypopharynx tumors, etc.). Four articles used data from the National Cancer Database (NCDB) at a similar period,^9^, ^10^, ^11^, ^12^ one could not be used because it had the same data seen in other articles.^9^ Three of them had different casuistics.^10^, ^11^, ^12^ and they were used in the metanalysis in different analyses. The same thing was done with the studies using the SEER database at similar times.^13^, ^14^, ^15^ So, 30 studies were used in the metanalysis (Table 1).^10^, ^11^, ^12^, ^13^, ^14^, ^15^, ^16^, ^17^, ^18^, ^19^, ^20^, ^21^, ^22^, ^23^, ^24^, ^25^, ^26^, ^27^, ^28^, ^29^, ^30^, ^31^, ^32^, ^33^, ^34^, ^35^, ^36^, ^37^, ^38^, ^39^

**Table 1:** List of articles used in the metanalysis, and data used for each article in the present study.

Study	Number of cases	Aspects evaluated
Bates et al., 2019^11^	11,010	OS-T3, OS-T4
Bussu et al., 2013^16^	166	OS, DSS, Dysp
Choi et al., 2016^17^	142	OS, OS-T4
Cocek et al., 2018^18^	185	Rec
Dyckhoff et al., 2017^19^	268	OS, OS-T4
Dziegielewski et al., 2012^20^	258	OS, OS-T3, OS-T4, DFS-T3
Foote et al., 2006^21^	101	Rec, Tube
Fuller et al., 2016^22^	412	Disf, Tube
Gourin et al., 2009^23^	264	OS-T4
Grover et al., 2015^24^	969	OS, OS-T4
Hsin et al., 2014^25^	62	Rec
Jones et al., 1992^26^	147	Rec
Karatzanis et al., 2014^27^	384	Rec
Lee et al., 2022^39^	237	DFS-T3, Rec, Dysp, Tube
Lin et al., 2016^14^	1935	OS, DSS,
Megwalu e Sikora, 2014^13^	5394	OS, DSS
Mulcahy et al., 2018^28^	548	OS, DSS
Nair et al., 2018^29^	120	OS, OS-T3, DFS-T3, Rec
Nocon et al., 2019^10^	3594	OS
Oh et al., 2019^30^	130	OS, OS-T4, DSS, Rec, Tube
O’Neil et al., 2016^15^	759	Tube.
Patel et al., 2011^31^	34	Rec
Patel et al., 2019^12^	6166	OS
Porter et al., 1998^32^	71	Rec
Reizenstein et al., 2014^33^	263	OS
Rosenthal et al., 2015^34^	221	Dysp, Tube.
Simpson et al., 1993^35^	74	OS, OS-T3, Rec
Spector et al., 2006^36^	49	Rec, Dysp, Tube
Timmermans et al., 2014^37^	182	OS, Rec
Vengalil et al., 2016^38^	107	OS, OS-T4, Tube

OS, Overall Survival; DSS, Disease Specific Survival; DFS, Disease-Free Survival; Rec, Recidive Index; Complic, Complications; Dysp, Dysphagia; Tube, Patients that need a feeding tube; Inc T2, Including T2-stage cases; T3, T3-stage cases; T4, T4-stage cases.

**Figure 2:**
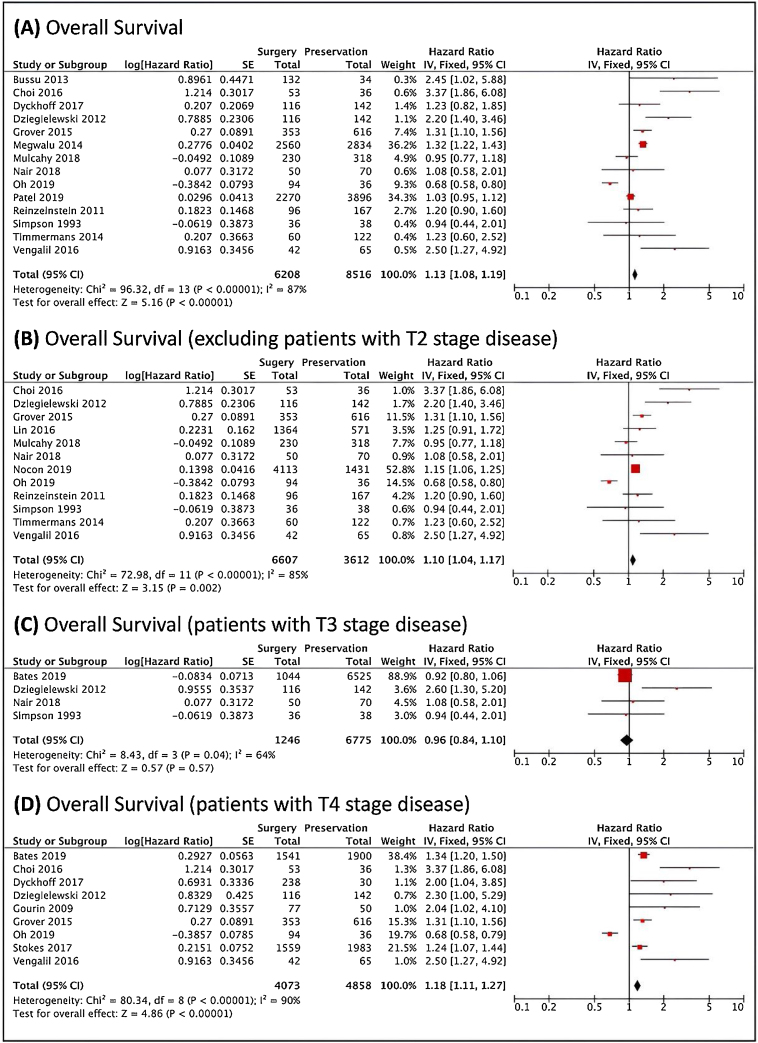
Survival analysis, analyzed through the risk of death. (A) Forest plot of the overall survival. (B) Forest plot of the overall survival, excluding the studies that included T2 patients. (C) Forest plot of the survival of patients with T3 local staging tumors. (D) Forest plot of the survival of patients with T4 local staging tumors. The Diamond on the right side shows a higher risk of death when Non-Surgical Treatment was used.

Fig. 2 shows survival analysis. There were better results with the Surgical Treatment (ST), since Non-Surgical Treatment (NST) was related to a higher risk of death. When excluding studies that included cases with local stage T2, this difference was maintained. When only T3 tumors were analyzed, four articles could be used. There were no significant differences in the reported results. In turn, in patients with T4 staging tumors, surgery was associated with better outcomes.

**Figure 3:**
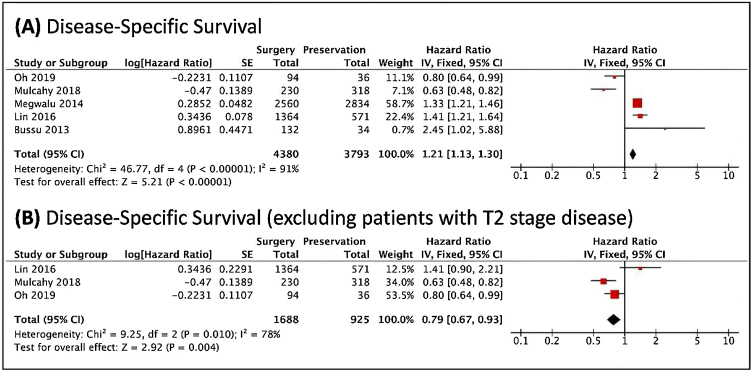
Forest plots of the disease-specific survival, analyzed through the risk of death, (A) in general. (B) When excluded T2 cases. The Diamond on the right side shows a higher risk of death when Non-Surgical Treatment was used. Diamond on the left side shows a higher risk of death when Surgery was used.

Regarding the disease-specific survival, TL was associated with better overall survival; however, when studies that contained patients with stage T2 tumors were removed, there was a lower risk of cancer death in patients undergoing the non-surgical treatment (Fig. 3). The analyses included very heterogeneous studies. It was not possible to perform stratification in patients with T3 and T4 tumors.

**Figure 4:**
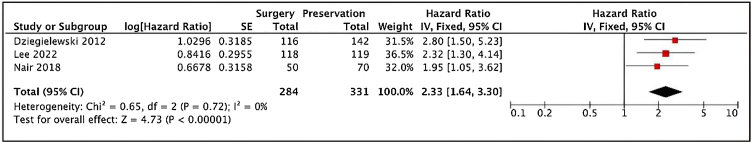
Forest plot of the disease-free survival of patients with T3 tumors. The Diamond on the right side shows a higher risk of recurrence or death when Non-Surgical Treatment was used.

It was possible to verify disease-free survival for T3 patients, and non-surgical treatment was related to a poorer outcome comparing to TL (Fig. 4).

**Figure 5:**
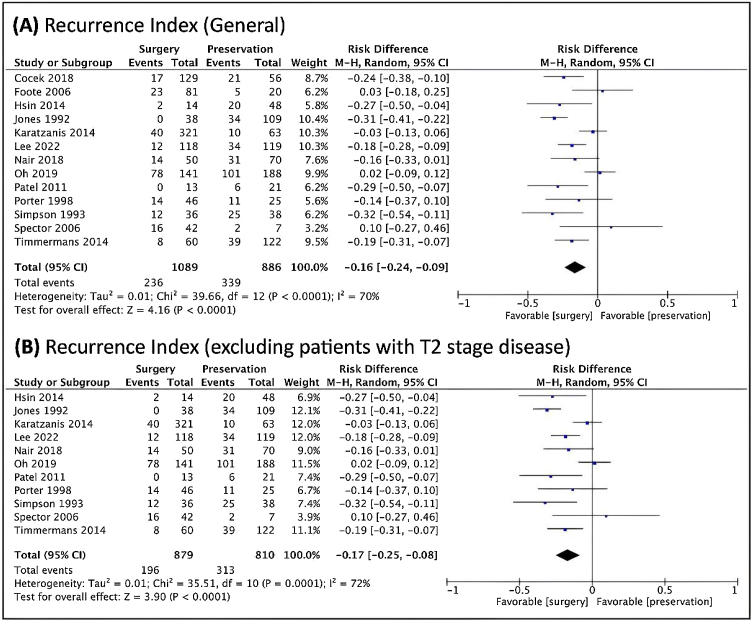
Forest plot of the recurrence index (A) in general. (B) when excluded T2 cases. The Diamond on the left side shows a lower recurrence risk when surgery was used.

TL was also better in relation to the risk of recurrence, both overall and when studies that included T2 patients were included (Fig. 5).

**Figure 6:**
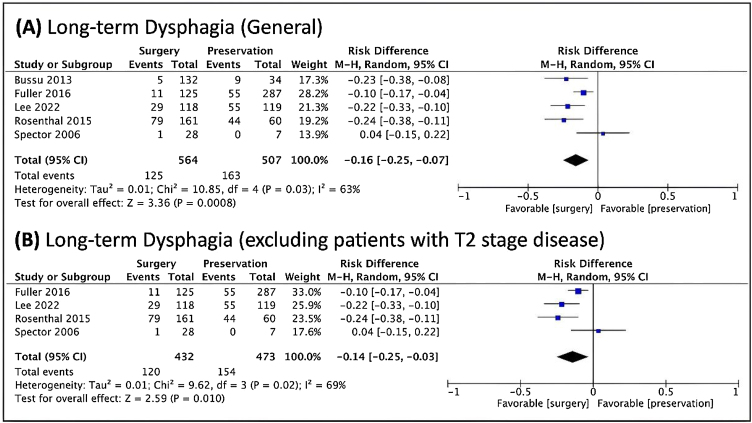
Forest plot of the prevalence of long-term dysphagia, (A) in general. (B) when excluded T2 cases. The Diamond on the left side shows a lower risk of long-term dysphagia when surgery was used.

**Figure 7:**
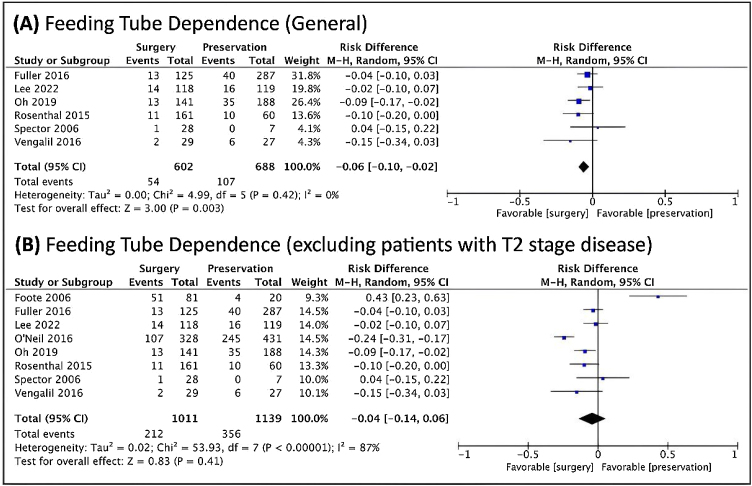
Forest plot of the prevalence of the feeding tube dependence, (A) in general. (B) when excluded T2 cases. The Diamond on the left side shows a lower risk of feeding tube dependence when surgery was used.

Regarding dysphagia and dependence on feeding tube, there was a relationship between the dysphagia and NST (Fig. 6), and a relationship between feeding tube dependence and the nonoperative treatment with the exclusion of T2 patients (Fig. 7).

## Discussion

Over the past decades, several studies have been conducted to define the best treatment for advanced SCC of the larynx. In the absence of prospective and randomized studies, a definitive conclusion on the best treatment modality is unlikely to exist.^40^ This study aims at a better elucidation to define the best option from the prognostic and functional point of view.

The Veterans Affairs (VA)^41^ study became the basis of most studies since it was published. It was the first prospective and randomized trial and demonstrated that 64% of patients undergoing NST had their larynges preserved, with no worse survival. After this study, comparable survival rates were found between surgical and non-surgical treatments, which led to a significant decrease in the number of TL performed over the years.^15^ The VA study included T2 patients within the inclusion criteria. So, the present study included this group of patients for a better comparison.

In 2006, worse laryngeal cancer survival rates were demonstrated, a trend contrary to what was happening to other types of cancer in the United States.^4^ From that moment on, the global tendency of using CRT to treat patients with advanced laryngeal carcinoma was questioned.^23^ Moreover, worse survival outcomes were demonstrated during periods when non-surgical treatments were being used more often.^33^

Several studies showed better survival rates when performing the surgical treatment, even including T2 patients.^10^, ^13^, ^16^, ^18^ On the other hand, other studies found no significant difference in these cases,^42^ even when T2 cases were excluded.^43^, ^44^

In our study, there was a higher risk of mortality when using NST as the initial treatment, even with the inclusion of patients with T2 stage. This shows that surgery remains the best treatment for patients with advanced laryngeal carcinoma in general, without other stratification.

The best survival rate obtained by the surgery is evident when we look at patients with more advanced local stage ‒ T4 cases. Several studies demonstrating this superiority of the TL in these cases have been published.^16^, ^19^, ^27^ In patients with T4a local stage with thyroid cartilage invasion, the primary treatment modality (surgery vs. CRT) was the only factor significantly associated with overall survival. So, surgery remains the key to the successful treatment of patients with T4 tumors, being considered the standard treatment for this type of patient.^8^, ^27^, ^34^

The main point of controversy involves patients with T3 stage tumors. Patients with T3 tumors have been the most indicated cases for the so-called non-surgical organ preservation therapy.^5^ In the study by Hoffman et al., the most notable deterioration in the prognosis over the two decades preceding the study occurred in T3 cases, due to the increased use of non-surgical or “organ preservation” therapies.^4^ Therefore, the use of this type of treatment would be associated with worse results when compared to the surgical treatment. In T3 tumors cases, while some studies showed a better survival with TL,^13^, ^16^, ^32^ other authors did not find a statistically significant difference.^36^ Beside, our study showed a better disease-free survival when TL was the treatment of T3 cases.

Regarding the non-surgical treatment, it was shown that concomitant CRT was superior to the induction CT followed by RT and to the exclusive RT in relation to larynx preservation and locoregional control, having similar survival rates among the three groups.^30^, ^45^ While there are studies showing that there is no change in survival when using CT combined with RT,^46^ others show a great advantage when there is such combination, both in overall survival^42^ and in the laryngectomy-free survival rate,^30^ including non-surgically treated T3 cases.^47^ So, in this metanalysis, when there were different groups of non-surgical treatment, it was decided to use the group of patients undergoing combined treatment, instead of the group undergoing exclusive RT. When there was only one group undergoing nonoperative treatment, this group was used.

In contrast, a metanalysis showed that, among the non-surgical treatments for patients with advanced laryngeal carcinoma, RT was the treatment with the best overall survival rate and disease-free survival. According to the authors, the combination of CT and RT could be associated with a higher toxicity, increasing mortality not directly related to the disease.^48^ This could explain the disease-specific survival results in our metanalysis. There is a relationship of better disease-specific survival in the non-surgical group when excluding T2 tumors, while the surgical group has better disease-specific survival rates when locally less advanced tumors are included. Including patients who did not receive CT in the treatment (case of less advanced tumors), the lower toxicity can lead to lower mortality from non-tumor causes, leading to a higher percentage of cancer deaths and, consequently, to a poorer disease-specific survival.

It has been described how the use of effect sizes and Confidence Intervals leads to better interpretations of results in different studies. Thus, the information can be used with a better comparison between the results, and their clinical effects are compatible. These aspects seen in the results are necessary for the meta-analytic thinking, for the best accumulation of knowledge from multiple studies.^49^ Thus, in this study, Hazard Ratios with Confidence Intervals were used in the metanalysis. Thereby, it is possible to have a better comparison of the survival rates between the different treatments, obtaining more reliable results.

There are not enough studies to perform a metanalysis to compare locoregional control. However, in the literature, we can see that surgery has an advantage over CRT,^22^, ^31^, ^34^, ^37^^,^^38^, ^42^, ^43^ sometimes without affecting the overall survival.^43^ This reflects a greater disease-free survival with TL.^20^

This better locoregional control is reflected in the lower recurrence rates observed in patients undergoing TL, as shown in this metanalysis. Authors who advocate the use of NST rely on patient survival. In many cases, survival and disease control rates with these treatments are because salvage surgery is frequently effective and is associated with good survival rates. Recurrent laryngeal cancer patients are considered the best candidates for salvage surgery among recurrent tumors in head and neck.^50^ But recurrence rate may be associated with worse survival rates. Tumor recurrence has been shown to be a major mortality factor in patients who have treated laryngeal carcinomas with CRT.^51^

Organ preservation has always been considered superior when analyzed the quality of life. However, more recent studies have had different conclusions. Despite affecting quality of life in different ways, TL and CRT may have similar life quality results. This may occur due to the evolution in rehabilitation techniques, especially in non-laryngeal voice acquisition;^52^ and to the aggressiveness of CRT treatment, leading to a high rate of esophageal and laryngeal dysfunction.^44^

When assessing the impacts of different treatments on the quality of life of head and neck cancer patients, the feeding tube is the strongest predictor of poor outcomes. Tracheotomy is a moderate factor, while TL is one of the weakest clinical variables to predict poor quality of life outcomes.^53^ So, long-term dysphasia and tube dependence were used for functional outcome in the present study. Most studies considered long-term dysphasia and tube dependence when the patient had symptoms or depended on tube feeding 6–12 months after treatment and had no prevision regarding any change about it.

Pretreatment dysphagia, CRT treatment, and salvage surgery can be considered high-risk predictors of long-term dysphagia, weight loss, gastrostomy use, and tube dependence.^54^ Thus, in patients with T4 tumors, treatment with CRT brings disappointing functional results.^53^ This may explain the results of feeding tube dependence found in this study. By using only studies with patients with T3 and T4 tumors (excluding T2), non-surgical treatment was associated with greater dependence on feeding tubes. By removing the less advanced cases, the cases with worse pre-treatment function were eventually used. The functional condition could be considered as a factor to explain worse survival associated with non-surgical treatments.

The objective of the non-surgical organ preservation treatment is a better function and quality of life, without compromising survival, for patients with advanced laryngeal carcinoma.^15^ This is possible in selected cases, especially in less advanced cases (T3) and patients able to receive the complete treatment. An important issue is that there is a lack of better criteria for selecting which patients would benefit from the non-surgical treatment in these cases.^12^

Over the last decade, some systematic reviews and metanalysis have been published comparing surgical treatment with non-surgical treatment in patients with advanced laryngeal carcinoma.^9^, ^10^, ^11^, ^12^, ^13^, ^14^, ^15^, ^16^, ^17^, ^18^, ^19^, ^20^, ^21^, ^22^, ^23^, ^24^, ^25^, ^26^, ^27^, ^28^, ^29^, ^30^, ^31^, ^32^, ^33^, ^34^, ^35^, ^36^, ^37^, ^38^, ^39^, ^40^, ^41^, ^42^, ^43^, ^44^, ^45^, ^46^, ^47^, ^48^, ^49^, ^50^, ^51^, ^52^, ^53^, ^54^, ^55^ One study showed that surgical treatment had superiority only in patients with T4 stage.^56^ Other studies with only T3 patients did not reach a conclusion about the best treatment between surgical and non-surgical treatment, since they presented similar survival outocomes.^55^, ^57^ A systematic review about the quality of life after advanced laryngeal carcinoma treatment did not reach a conclusion regarding the best treatment concerning this topic, with no difference in quality of life between the types of treatment.^58^ A systematic review with metanalysis published in 2018 showed advantage of ST only in T4 patients among surgical patients, and T3 patients did not have worse survival when treated with organ preservation therapy.^59^ Many articles used in this metanalysis were not included in our study due to insufficient data for inclusion. Among the cited articles, this was the only one using Hazard Ratio and confidence intervals in the metanalysis.

Some affairs must be considered when doing such review. The way the studies were conducted and how the patients were distributed among the different treatments should always be pointed. Sometimes there is not a proper selection due to the lack of randomization,^33^, ^37^ or even a better standardization of the treatments, which may produce different, and consequently less reliable results.^12^, ^30^ Some studies eventually included patients who have not received a curative dose of RT, while other studies included patients that have not received associated CT.^11^, ^13^, ^14^, ^17^^,^^24^, ^34^ This lack of uniformity in the various studies is reflected in the absence of a better homogeneity found in our metanalysis. As the various studies did not use uniform criteria, it will be difficult to achieve a truly homogeneous review.

Otherwise, there are major limitations when comparing different treatment methods using retrospective data, as randomized trials are the gold standard for detecting differences in outcomes while minimizing bias. The Veterans Affairs study is practically the only prospective randomized trial including a surgical group. It is very unlikely that any future study with these characteristics will be made, due to ethical issues and patient and physician preferences.^52^ Thus, this metanalysis has a number of factors that may lead to poorer results: first, the lack of prospective and randomized studies comparing the treatment options in cases of advanced laryngeal cancer; second, the lack of standardization in the studies, mainly because they are not prospective studies and there is no real consensus on how the treatment should be performed; and, the lack of data contained in several papers, reducing the number of studies that could be included in the analysis. Even so, it can be confirmed that, in patients with advanced tumors in general, non-surgical treatment is related to higher mortality risk. The same conclusion cannot be reached for patients with T3 tumors when they are studied separated. Nevertheless, from a recurrence and functional point of view, TL has better results when compared to non-surgical organ preservation treatment.

## Conclusion

In general, patients with advanced laryngeal SCC have better surviving rates when submitted to TL as the first-choice treatment. Patients with T4 tumors should have TL as their treatment of choice. In patients with T3 tumors there are similar survival rates with both treatments. Nonetheless, when using NST, there is a higher chance of recurrence and, consequently, the need for salvage laryngectomy. In addition, in the functional aspect, dysphagia and feeding tube dependence are more likely to happen when using this kind of treatment. Thus, it is important to note that organ preservation treatment of the larynx does not always mean the actual preservation of a functioning organ.

## Conflicts of interest

None of the authors have received any funding and all authors declare no conflict of interest.
